# Arsenite Selectively Inhibits Mouse Bone Marrow Lymphoid Progenitor Cell Development In Vivo and In Vitro and Suppresses Humoral Immunity In Vivo

**DOI:** 10.1371/journal.pone.0093920

**Published:** 2014-04-08

**Authors:** Peace C. Ezeh, Fredine T. Lauer, Debra MacKenzie, Shea McClain, Ke Jian Liu, Laurie G. Hudson, A. Jay Gandolfi, Scott W. Burchiel

**Affiliations:** 1 Department of Pharmaceutical Sciences, College of Pharmacy, University of New Mexico, Albuquerque, New Mexico, United States of America; 2 Department of Pharmacology and Toxicology, College of Pharmacy, University of Arizona, Tucson, Arizona, United States of America; The University of Arizona, United States of America

## Abstract

It is known that exposure to As^+3^ via drinking water causes a disruption of the immune system and significantly compromises the immune response to infection. The purpose of these studies was to assess the effects of As^+3^ on bone marrow progenitor cell colony formation and the humoral immune response to a T-dependent antigen response (TDAR) *in vivo*. In a 30 day drinking water study, mice were exposed to 19, 75, or 300 ppb As^+3^. There was a decrease in bone marrow cell recovery, but not spleen cell recovery at 300 ppb As^+3^. In the bone marrow, As^+3^ altered neither the expression of CD34+ and CD38+ cells, markers of early hematopoietic stem cells, nor CD45−/CD105+, markers of mesenchymal stem cells. Spleen cell surface marker CD45 expression on B cells (CD19+), T cells (CD3+), T helper cells (CD4+) and cytotoxic T cells (CD8+), natural killer (NK+), and macrophages (Mac 1+) were not altered by the 30 day *in vivo* As^+3^ exposure. Functional assays of CFU-B colony formation showed significant selective suppression (p<0.05) by 300 ppb As^+3^ exposure, whereas CFU-GM formation was not altered. The TDAR of the spleen cells was significantly suppressed at 75 and 300 ppb As^+3^. *In vitro* studies of the bone marrow revealed a selective suppression of CFU-B by 50 nM As^+3^ in the absence of apparent cytotoxicity. Monomethylarsonous acid (MMA^+3^) demonstrated a dose-dependent and selective suppression of CFU-B beginning at 5 nM (p<0.05). MMA^+3^ suppressed CFU-GM formation at 500 nM, a concentration that proved to be nonspecifically cytotoxic. As^+5^ did not suppress CFU-B and/or CFU-GM *in vitro* at concentrations up to 500 nM. Collectively, these results demonstrate that As^+3^ and likely its metabolite (MMA^+3^) target lymphoid progenitor cells in mouse bone marrow and mature B and T cell activity in the spleen.

## Introduction

Arsenic occurs ubiquitously in nature as an environmental pollutant found in the air, soil and water. The pervasive nature of arsenic pollution makes it a major concern globally despite environmental regulations in some countries. Humans are exposed to some level of arsenite (As^+3^) via drinking water, food crops and air, and exposure is associated with many diseases [1]. The US EPA set the maximum contaminant level (MCL) for arsenic in drinking water at 10 ppb. However, in many parts of the US and elsewhere in the world, people rely on private unregulated wells for their drinking water supply and therefore are exposed to higher levels of arsenic. Levels exceeding 500 ppb have been found in domestic well water in some parts of the US [2]. Humans are exposed to two primary forms of inorganic arsenic: trivalent arsenic (As^+3^) and pentavalent arsenic (As^+5^). Both of the forms undergo biotransformation involving reduction and oxidative methylation in the liver, to form methylated arsenicals such as MMA^+5^, MMA^+3^, DMA^+5^, and DMA^+3^ [3], [4], [5]. MMA^+3^ is thought to be the most toxic arsenic species *in vitro* [6].

Previous studies have shown that exposure to arsenic via drinking water alters components of the innate and adaptive immune system in mouse lung and significantly compromises the immune response to infection [7]. Several mechanisms including interruption of cell signaling, altered expression of transcription factors, oxidative stress due to formation of reactive oxygen species, increased apoptosis, chromosomal aberration and inhibition of DNA repair and poly(ADP-ribose) polymerase (PARP) activity have been proposed for arsenic toxicity [8], [9], [10], [11], [12], [13], . The bone marrow is a vital organ of the immune system in which all immune cells originate through the process of hematopoiesis. In order to elucidate the role and mechanism of arsenic in immune suppression, it is necessary to study the effects of environmentally relevant levels of arsenic on the bone marrow and peripheral lymphoid organs. Hematopoietic stem cells are able to commit to specific lineages in response to signal(s) from the microenvironment using the expressed receptor(s) [16]. Modulation of the bone marrow microenvironment by arsenic may alter stem cell lineage populations. The pre-B and granulocyte-monocyte colony forming unit (CFU-B and CFU-GM) assays are indicators of the bone marrow lymphoid and myeloid responses to immunotoxicity and may define an aspect of immunity that may be compromised. Studies show that arsenic exposure impacts the immune system or alters immune responses in different ways. In humans, experiments show that chronic arsenic exposure causes decrease in T-cell proliferation and cytokine secretion [17], impairment of macrophage functions [18], and increased apoptosis of peripheral blood mononuclear cells [19]. Given the association of arsenic exposure to immunosuppression, hematotoxicity, and associated diseases, one would expect that the immunosuppressive role of arsenic and its metabolites should be evident on the development of the progenitors as well as peripheral lymphoid organ activity.

Previous studies in our laboratory show that *in vitro* As^+3^ exposure produced immunosuppression at concentrations as low as 500 nM in mouse spleen cells [20]. The direct effect of As^+3^ and MMA^+3^ on bone marrow progenitor cells has not been evaluated. To investigate the *in vivo* effects of As^+3^, murine bone marrow and spleen cells were examined following a 30 day oral exposure of mice. CFU-B formation and spleen cell TDAR activity were suppressed by As^+3^. The effects of direct exposure of As^+3^, MMA^+3^, and As^+5^ on *in vitro* progenitor cell cultures was monitored. Collectively, our results show that As^+3^ and MMA^+3^ selectively suppress the formation of lymphoid progenitors in murine bone marrow *in vitro*, demonstrating that lymphoid cell differentiation in the bone marrow and lymphoid cell activity in the spleen are important targets of arsenic action.

## Methods

### Animals

C57BL/6J male mice were purchased at 8 to 10 weeks of age from Jackson Laboratory (Bar Harbor, ME) and were acclimated in our AAALAC-approved animal facility for at least one week before being used in experiments. All animals were handled in accordance to procedures and protocols approved by the Institutional Animal Use and Care Committee at the University of New Mexico Health Sciences Center. Following acclimation, mice (2–3 per cage) were exposed to arsenite (As^+3^) at different concentrations in parts per billion (ppb) for 30 days via drinking water. Stock solutions were prepared using sterile double processed tissue culture water from

Sigma-Aldrich. Drinking water bags were weighed to determine the amount of water consumed by each group of 2–3 mice. Drinking water contained less than 5 ppb total arsenic. For the *in vivo* experiments, the concentration of arsenic in drinking water was periodically checked and validated by the University of Arizona Laboratory for Emerging Contaminants (ALEC, Tucson AZ). Mouse 2020X Teklad Global Soy Protein-Free Extruded Rodent Diet, Harlan Laboratories Inc, Madison Wisconsin, USA, www.harlan.com contained 0.16 mg/kg (160 ppb) of total arsenic, of which approximately 10–15% may be considered to contain inorganic arsenic species [21].

Each exposure or treatment group consisted of five mice and bone marrow cells from each mouse were analyzed in triplicate. *In vitro* studies were performed using bone marrow cells pooled together from three untreated C57BL/6J male mice femurs and each treatment was run in triplicate. All animals exposed to these chemicals were handled with caution, using personal protective equipment, and disposed in accordance with the University of New Mexico's Risk and Safety Committee and the State of New Mexico guidelines.

### Chemicals and Reagents

Sodium arsenite (CAS 774-46-5, Na AsO2) and sodium arsenate dibasic heptahydrate (CAS 10048-95-0, Na2HAsO4.7H2O) were purchased from Sigma-Aldrich (St. Louis, MO). Monomethylarsonous acid (MMA^+3^) >98% purity, was prepared by the Synthesis Core of the Southwest Environmental Health Sciences Center at the University of Arizona (Tucson, AZ). MethoCult GF methylcellulose medium (Cat. No. M3534) with recombinant cytokines (without EPO) for mouse cells was purchased from Stem Cell Technologies (Vancouver, BC, Canada). Mouse methylcellulose complete media for pre-B cells (Cat. No. HSC009) was purchased from R&D Systems (Minneapolis, MN).

### Isolation of Mouse Bone Marrow Cells

Bone marrow cells were isolated according to the procedure outlined in the Stem Cell Technologies Technical Manual version 3.1.1(http://www.stemcell.com/). Briefly, each mouse was sacrificed and 70% isopropyl alcohol was immediately used to wet the ventral fur to avoid contamination at site of dissection. Both femurs were collected sterilely and placed and held on ice in Hanks' Balanced Salt Solution (HBSS) purchased from Lonza (Walkersville, MD). To extract cells, femurs were placed in petri dish containing cold sterile RPMI 1640 Medium supplemented with 2% Fetal Bovine Serum (FBS). The ends of the femurs were trimmed to expose interior marrow shaft. Using a 1 cc syringe with a 25 gauge needle, approximately 1 ml cold sterile medium was flushed through the femur several times to release cells into a petri dish. The medium containing cells from both femurs was immediately transferred to a 15 ml culture tube and placed on ice until needed. Cells were washed via centrifugation at 4°C, 400 × g for 10 min and were resuspended in RPMI media (as above) for culturing or flow cytometry analysis as discussed below. Cell viability was determined by Acridine Orange/Propidium Iodide (AO/PI) staining and counting using the Nexcellom Cellometer 2000.

### Isolation of Mouse Spleen Cells

Spleen cells were isolated following the procedure described by Lauer et al [22]. Briefly, harvested mouse spleens were weighed and placed in HBSS. Cells from each spleen were isolated and homogenized by placing the organ between the frosted ends of two microscopic glass slides and squeezing into a dish containing media. The cell suspension was centrifuged at 280×g and 4°C for 10 min. The cell pellets were collected and resuspended in complete media containing RPMI 1640 (Sigma-Aldrich) w/10% FBS (Hyclone Logan, UT), 2 mM L-glutamine (GIBCO by Life Technologies, Grand Island, NY), 100 μg/ml streptomycin, 100 units/ml penicillin (GIBCO by Life Technologies) and placed on ice. Cell viability was determined by acridine orange/propidium iodide (AO/PI) staining and counting using the Nexcellom Cellometer 2000.

### Bone Marrow and Spleen Cell Surface Markers by Flow Cytometry

For bone marrow cell surface marker subset analyses, antibody cocktails for CD34, CD38, CD45 and CD105 were obtained from BD Biosciences. CD34 and CD38 define early stages of hematopoietic cell development [23] and CD105 defines a population of CD45- mesenchymal stem cells and stromal cells [24]. Spleen cell surface markers were analyzed using a custom cocktail obtained from BD Biosciences (San Jose, CA) for CD45, CD3, CD4, CD8, CD19, NK and Mac1 for spleen cells as previously described, [22]. The control cocktail for the BM included isotype controls and CD45-PerCP (IgG2a-FITC; IgG2a-PE, CD45-PerCP;IgG2a-Alexa647), and the test cocktail included CD34-FITC; CD38-PE; CD45-PerCP; CD105-Alexa647. Briefly, 100 μl of media containing 1×10^6^ bone marrow cells were incubated with 20 μl of cocktail for 30 min at RT in the dark. Red blood cells were lysed by incubating with 2 ml of ammonium chloride lysing solution (0.15 M ammonium chloride, Sigma A-4514; 10 mM sodium bicarbonate, Sigma S-5716; 1 mM disodium EDTA, Sigma-E7889; pH 7.4) for 10 min. The cells were centrifuged at 275×g for 10 min, the supernatant was aspirated and the cells washed with 2 ml Dulbecco's phosphate buffered saline (DPBS) w/o Ca^+2^ and Mg^+2^ containing 1% FBS and 0.9% sodium azide (PBS/FBS). The washed cells were resuspended in 0.5 ml PBS/FBS and 20,000 cells were analyzed using the Accuri C6 Flow Cytometer (Becton Dickinson, San Jose, CA). The percent positive cell population was determined by gating on CD45+ cells and subtracting out background determined from the isotype controls for each sample. Spleen cell surface markers were analyzed as previously described [22], using custom cocktail from BD Biosciences containing antibody reagents for CD3(FITC)/CD8a(PE)/CD45(PerCP)/CD4(APC) or CD3+CD19(FITC)/PanNK(PE)/CD45(PerCP)/Mac-1(APC).

### T-Dependent Antibody Assay

For the T-dependent antibody response (TDAR) assay, mice were immunized with 0.2 ml of 10% sheep red blood cells (Colorado Serum, Denver CO) in saline 4 days prior to euthanization. Following spleen harvesting, cells were washed and suspended at 4×10^6^ cell/ml in supplemented media. A mixture of 100 μl cells (4×10^5^), 50 μl washed 1% sheep red bloods cells and 400 μl of 0.8% low-melting point SeaPlaque agarose (Lonza, Rockland, ME) solution (in RPMI held at 42°C) were combined and poured onto an agarose coated slide. Agarose was allowed to set-up before incubating face down on custom slide trays in a humidified plastic box at 37°C for 1.5 hour. Guinea pig complement (Colorado Serum, Denver, Colorado) was diluted 1∶20 in Dulbecco's Phosphate Buffered Saline containing Ca^2+^ and Mg^2+^ (Sigma) and warmed to room temperature. Slides were flooded with diluted complement following the 1.5 hour incubation and then incubated, as described above for an additional 2 hrs. Slides were removed from the incubator and stored in a cold 0.85% sodium chloride solution. SRBC lysis was quantified by counting plaques in the SRBC/agar lawn using a dissecting microscope. Results are expressed as plaque forming cells (PFC) per culture of 4×10^5^ cells.

### CFU-B Assay

This assay was performed as described in Stem Cell Technologies Technical Manual version 3.1.1 (http://www.stemcell.com/) for Mouse Colony-Forming Cell (CFC) Assays using MethoCult. Briefly, isolated bone marrow cells from each mouse (for *in vivo* studies) or pooled from three mice (for *in vitro* studies) were suspended in RPMI 1640 Medium supplemented with 2% heat inactivated Hyclone Fetal Bovine Serum (Fisher Scientific, Pittsburgh, PA) at 1×10^6^ cells/ml. 400 μl (4×10^5^ cells) of the cell suspension was transferred to a 16 ml (17×100 mm) sterile culture tube which contained 4 ml Mouse Methylcellulose Complete Media for Pre-B Cells (R&D Systems, Minneapolis, MN).The tube was thoroughly mixed by vortexing and allowed to sit for 20 min to release air bubbles. 1 ml of the methylcellulose-cell mixture was dispensed into a 35 mm culture dish (Stem Cell Technologies) using a 3 cc syringe with a 16G×11/2″ Monoject Aluminum Hub, blunt canula needle from Covidien (Mansfield, MA).Samples were run in triplicate. The mixture was evenly dispersed in the dish by rocking the dish. One sterile water dish and two sample dishes were placed in a 100 mm culture dish and incubated at 37°C, 5% CO_2_, in a humidified incubator for 10 days. CFU-B colonies were counted and recorded for statistical analysis.

### CFU-GM Assay

The same initial methods as in the CFU-B assay described above were used for the CFU-GM assay except that the isolated bone marrow cells were suspended in Iscove's Modified Dulbecco's Medium (Sigma-Aldrich) supplemented with 2% heat inactivated Hyclone Fetal Bovine Serum at 2×10^5^ cells/ml. 400 μl (8×10^4^ cells) of the cell suspension was transferred to a 16 ml (17×100 mm) sterile culture tube which contained 4 ml MethoCult GF Methylcellulose Medium (Stem Cell Technologies). As described above for CFU-B, the mixture was evenly dispersed in the dish by rocking the dish. Sample dishes (35 mm) were placed in a 100 mm culture dish and put in the humidified incubator at 37°C and 5% CO_2_ for 14 days. CFU-GM colonies were counted and recorded for statistical analysis.

### Annexin V Staining

Bone marrow cells from femurs of three male C57BL6/J mice were pooled together and incubated in RPMI media supplemented with 2% FBS, 2 mM L-glutamine, 100 units/ml penicillin and 100 units/ml streptomycin with treatment for 18 hrs. Following incubation the cells were washed in PBS by centrifuging at 300×g. Cells were resuspended and stained in solutions and reagents provided in the FITC Annexin V Apoptosis Detection kit (BD Biosciences) according to manufacturer's procedure. Stained cells were analyzed using the Accuri C6 Flow Cytometer. Cells were selected using FSC and SSC and then analyzed based on percentage of stained cells.

### Data Analysis and Statistics

Data were analyzed using the Sigma Stat version 3.5 and Sigma Plot 12.0 software, one-way analysis of variance (ANOVA) and Dunnett's t-test to determine differences between control and treatment groups. For *in vivo* studies, treatment groups consisted of five animals and each animal was analyzed in triplicate. Bone marrow cell recovery was expressed as the mean number of recovered bone marrow cells obtained from a pool of both femurs. In the *in vitro* experiments, a treatment group consisted of three replicates of one chemical treatment of pooled bone marrow cells or control. We calculated the number of CFU-B and CFU-GM formed per 10^6^ cells plated at the beginning of the culture periods.

## Results

C57BL/6J male mice (5 per group) were given 0, 19, 75, or 300 ppb As^+3^ via drinking water for 30 days. As shown in Table 1, at the end of the 30 day period, there were no significant differences in body weights or spleen weights between groups and arsenic contained in the drinking water did not affect the amount of water consumed by the mice. There was an apparent trend towards a dose-dependent reduction in bone marrow cell recovery, which was significant at the 300 ppb As^+3^ exposure level (p<0.05). Spleen cell recovery was not affected by As^+3^ at any exposure level.

**Table 1:** 30 Day Arsenic Drinking Water Exposure and Effects on Mouse Body Weight, Spleen Weight, Bone marrow Cell and Spleen Cell recovery^1^

Arsenite treatment	Water consumed (ml)	Mean wt. of mice (g)	Mean Spleen wt. (g)	Mean BM cell recovery	Mean Spleen cell recovery
Control	125.1+29.2	29.2+1.0	0.107+0.01	3.13+.64 E+07	1.63+.29 E+08
19 ppb	112.2+13.4	29.3+2.0	0.110+0.02	3.22+.36 E+07	1.89+.45 E+08
75 ppb	136.0+20.7	28.2+2.0	0.10+0.02	2.62+.28 E+07	2.05+.39 E+08
300 ppb	148.3+13.8	29.0+2.0	0.11+0.02	2.46+.41 E+07*	1.63+.20 E+08

1 Five mice were examined for each group; results are Means + SD with statistical significance at *p≤0.05

**Figure 1:**
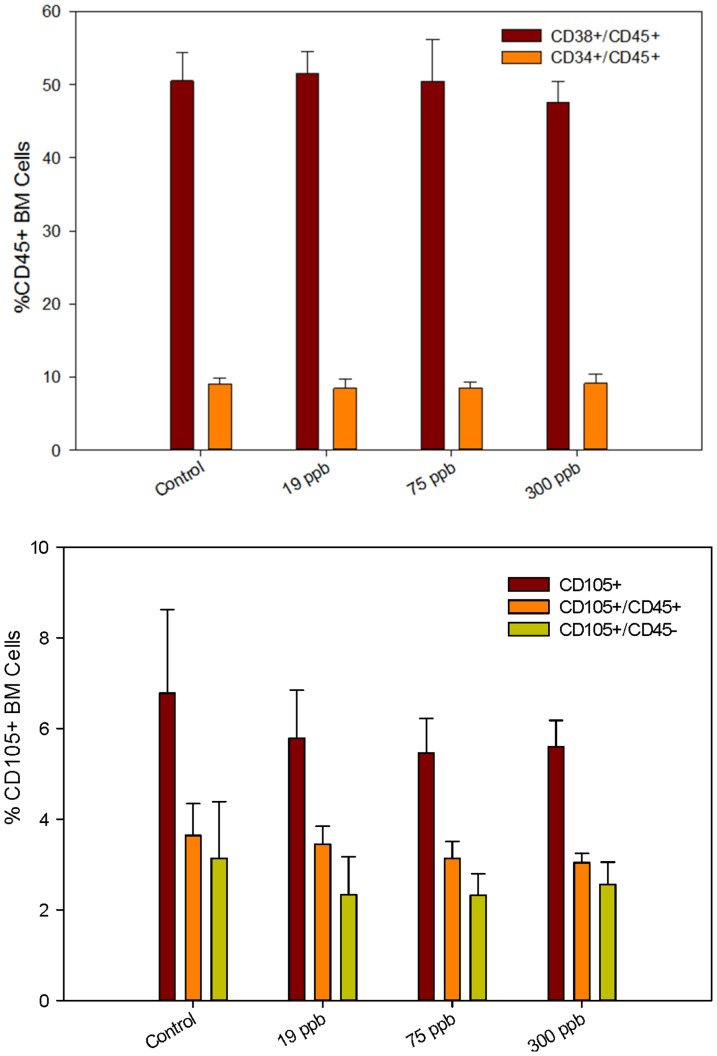
Bone marrow (BM) cell surface marker expression analyzed by flow cytometry. Percent CD34+ and CD38+ mice BM cells co-expressing CD45 [Top] and percent BM cells expressing CD105 [Bottom] in CD45+ and CD45- cells after 30 day consumption of As^+3^ in drinking water. Results are Means + SD.

**Figure 2:**
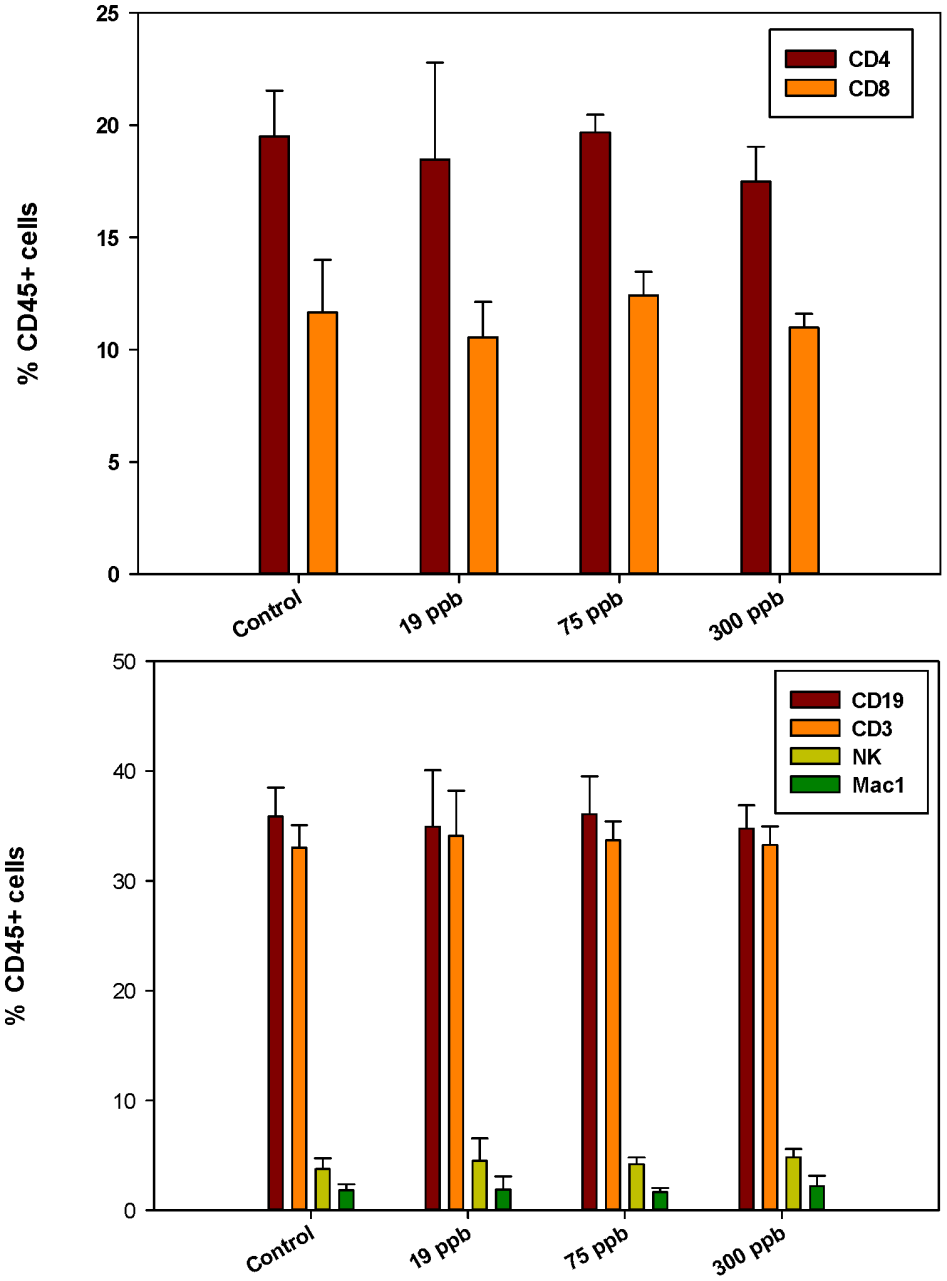
Spleen cell surface marker expression analysed by flow cytometry. Percent CD19+, CD3+, NK and Mac 1 cells co-expressing CD45 [Top] and percent CD4+ or CD8+ cells co-expressing CD45 [Bottom] after 30 day consumption of As^+3^ in drinking water. Results are Means + SD

Flow cytometry analysis of the bone marrow cell surface markers demonstrated that the percentage of early stem cells CD45+/CD34+ and CD45+/CD38+ (which define early hematolymphoid cells, [23]) was not changed by As^+3^ consumption (Figure 1, top). Also, there was no significant difference in CD45−/CD105+ (markers of mesenchymal stem cells, [24]) expression between control and treatment groups (Figure 1, bottom). In the spleen, there were no differences in cell surface marker expression for mature B (CD45+/CD19+), T (CD45+/CD3+), helper T (CD45+/CD4+), cytotoxic T (CD45+/CD8+), natural killer (CD45+/NK+), and macrophage (CD45+/Mac-1+) populations between control and treatment groups (Figure 2, Top and bottom).

**Figure 3:**
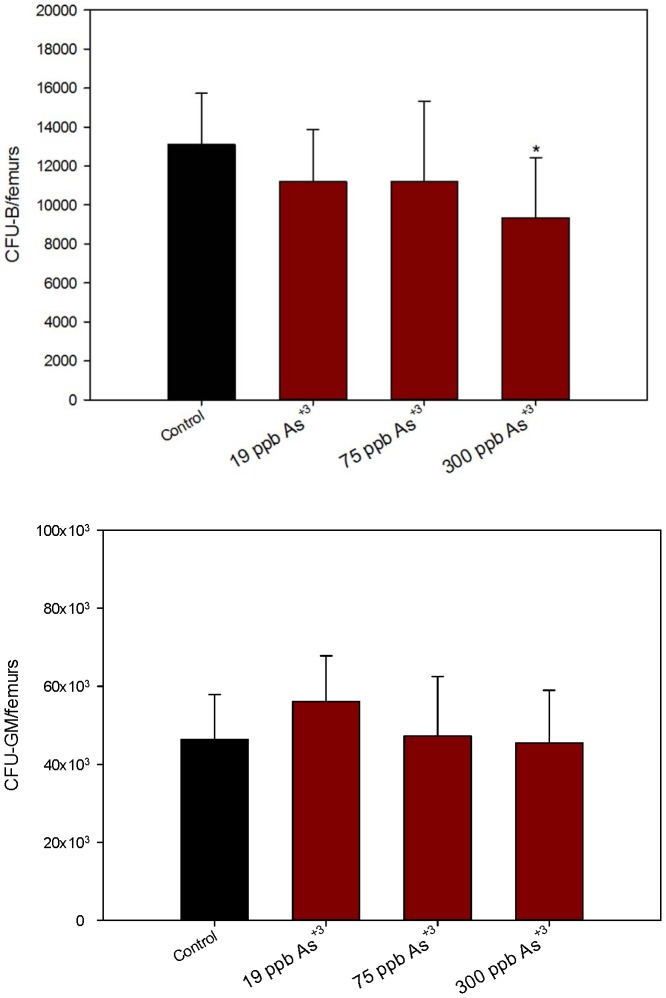
*In vivo* BM exposure to As^+3^ showing number of colonies per pair of mice femurs. Number of CFU-B colonies in a pair of mice femurs, 10 days post plating in mouse methylcellulose media for pre-B [Top]. Number of CFU-GM colonies in a pair of mice femurs 14 days post plating in Methocult media for GM selection [Bottom] after 30 day consumption of As^+3^ via drinking water. *Significantly different compared to control (p<0.05). Results are Means + SD.

**Figure 4:**
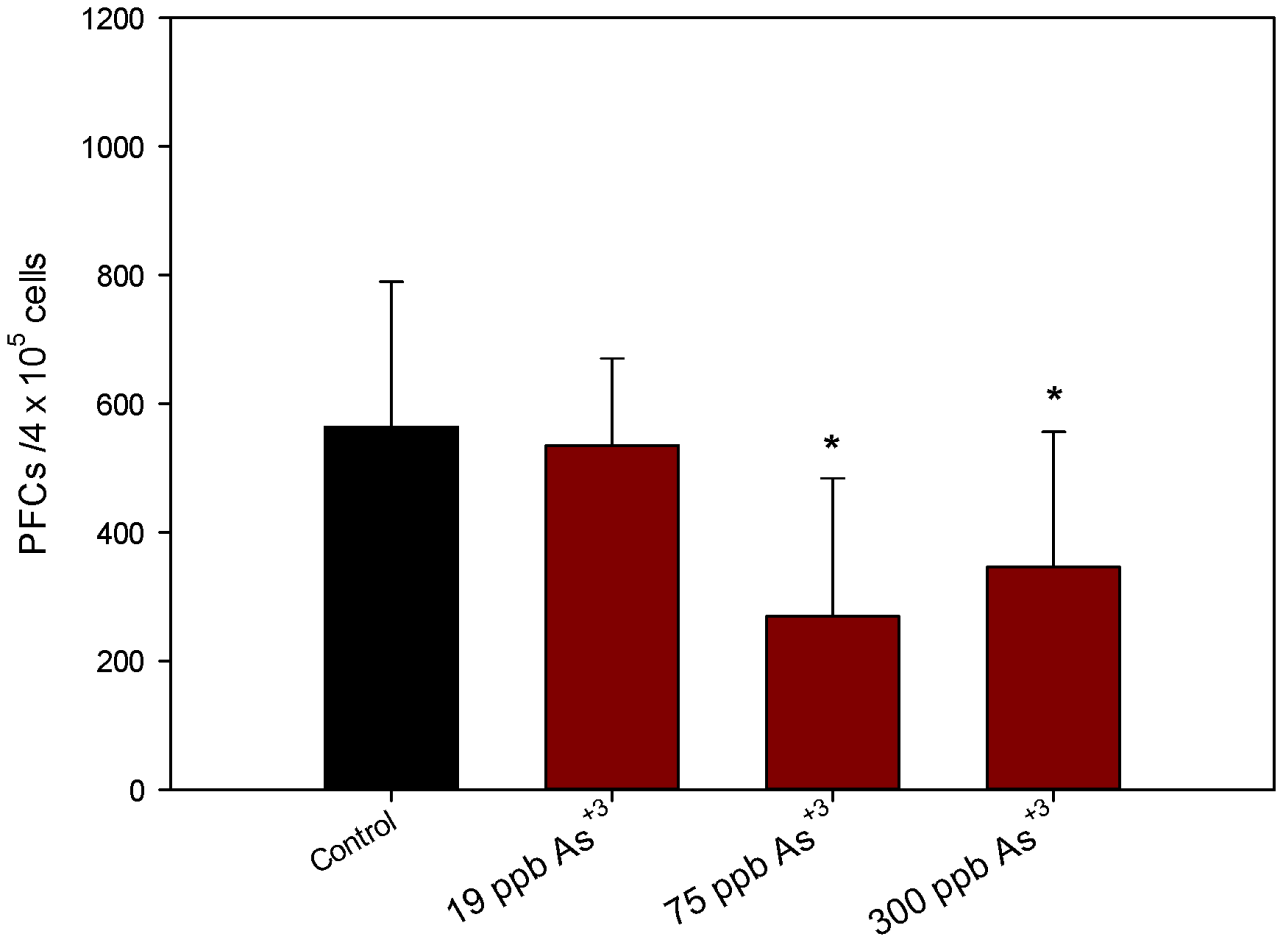
Suppression of T-dependent antibody response to sheep red blood cells post 30 day *in vivo* exposure of mice to As^+3^ via drinking water. After As^+3^ exposure, mice were immunized with SRBCs and examined for *in vitro* antibody production 4 days later. Results are Means + SD.

Since the purpose of these studies was to assess the effects of As^+3^ on lymphoid cell differentiation and function, we examined the colony forming unit (CFU) activity of pre-B cells and granulocyte-monocyte (GM) CFUs as well as peripheral lymphoid (spleen) cell function. As^+3^ was found to decrease the numbers of CFU-B measured in the femurs of the 300 ppb group (Figure 3, top), but it did not affect the number of CFU-GM detected (Figure 3, bottom). These results show a selective loss in lymphoid CFU activity, but not myeloid activity without altering the early progenitors (as measured by CD34/CD38) detected by flow cytometry. The T-dependent antibody response to sheep red blood cells was suppressed at both the 75 and 300 ppb exposure levels in the spleen (Figure 4) suggesting that immature and mature lymphoid lineages are sensitive to the effects of As^+3^.

**Figure 5:**
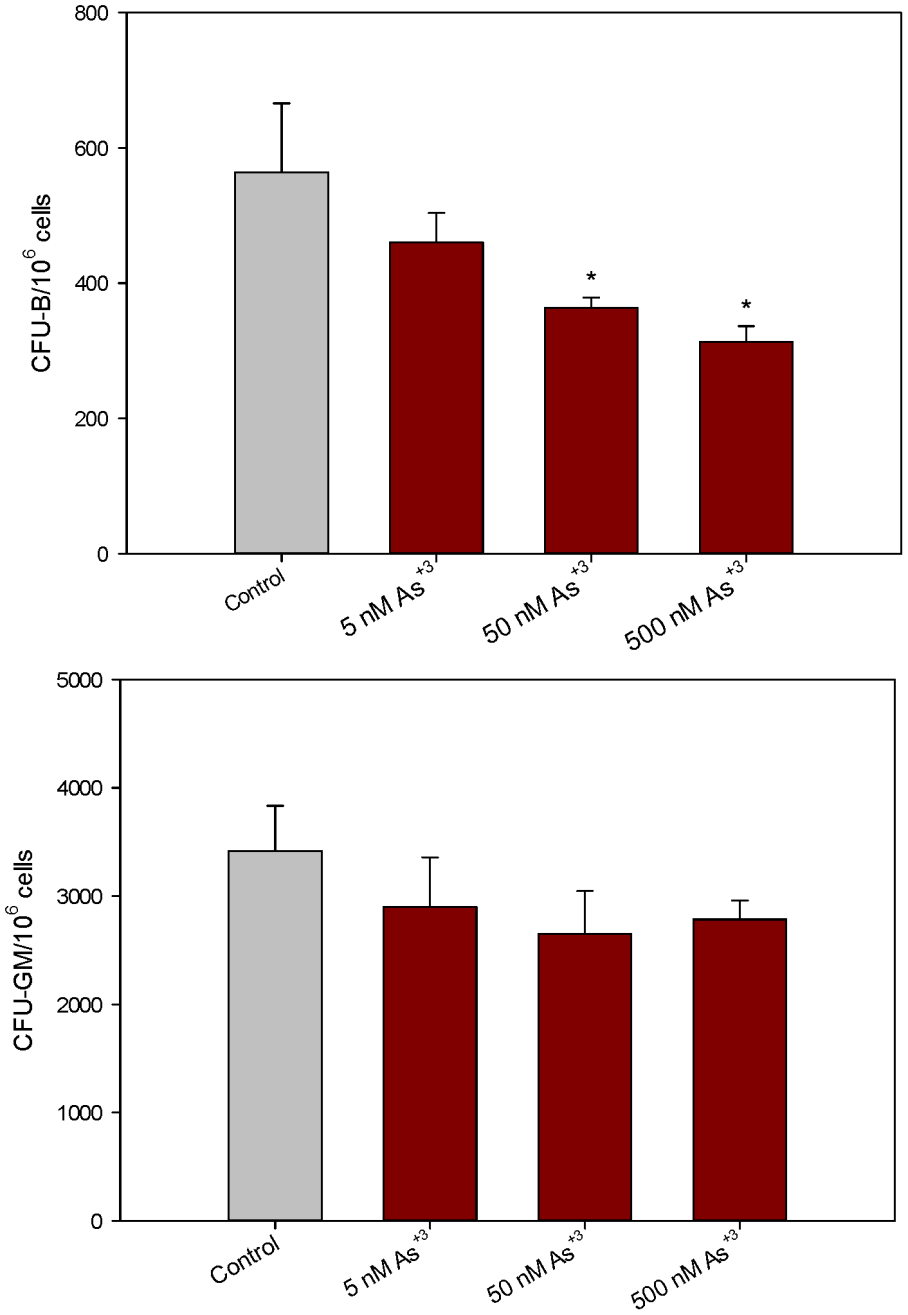
Number of colonies per million cells exposed to As^+3^ *in vitro*. Number of CFU-B colonies per million cells 10 days post plating in mouse methylcellulose media (containing As^+3^) for pre-B cells [Top]. Number of CFU-GM colonies per million cells 14 days post plating in mouse methylcellulose media (containing As^+3^) for GM cells [Bottom]. * Significantly different compared to control (p<0.05). Results are Means + SD.

To determine if As^+3^ has direct effects on bone marrow progenitor cells, we performed *in vitro* studies with known concentrations of As^+3^ over a wide concentration range. These studies also allowed us to examine the effects of MMA^+3^ on lymphoid and myeloid progenitor cultures, which is important because MMA^+3^ is a key and oftentimes more toxic metabolite of arsenite. In agreement with our *in vivo* studies, we found that pre-B cells were more sensitive than CFU-GM progenitors to *in vitro* exposure to As^+3^ (Figure 5). As^+3^ was found to suppress CFU-B formation at concentrations as low as 50 nM, whereas, this concentration and 500 nM As^+3^ did not affect CFU-GM formation.

**Figure 6:**
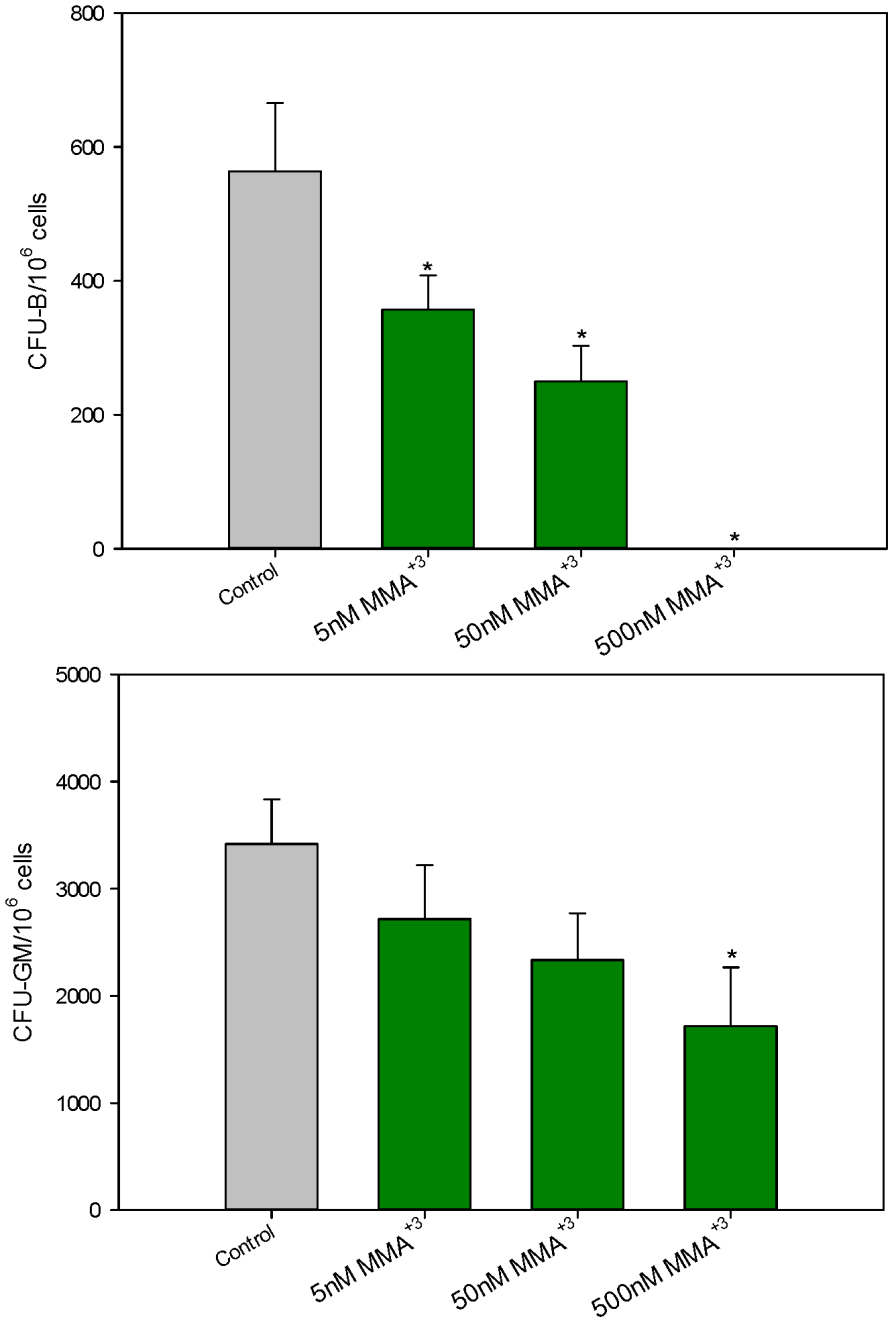
Number of colonies per million cells exposed to MMA^+3^ *in vitro*. Number of CFU-B colonies per million cells 10 days post plating in mouse methylcellulose media (containing MMA^+3^) for pre-B cells [Top]. Number of CFU-GM colonies per million cells 14 days post plating in mouse methylcellulose media (containing MMA^+3^) for GM cells [Bottom]. *Significantly different compared to control (p<0.05). Results are Means + SD.

Exposure of murine bone marrow cells to MMA^+3^ was also found to selectively suppress pre-B colony formation. There was a concentration-dependent suppression of CFU-GM by MMA^+3^ (Figure 6, bottom). At concentrations as low as 5 nM MMA^+3^, CFU-B formation was significantly suppressed (Figure 6, top), suggesting that MMA^+3^ may be approximately ten times more potent than As^+3^ in suppressing CFU-B formation. MMA^+3^ suppression of CFU-GM formation was significant at a concentration of 500 nM (Figure 6, bottom). The *in vitro* exposure to 500 nM MMA^+3^ proved to be nonspecifically cytotoxic to bone marrow cultures, as revealed by cell viability and Annexin V studies performed at 18 hrs after exposure (Table 2). Annexin V positive cells were found with both propidium iodide positive and negative cell populations, suggesting a mixed mode of cell death.

**Table 2:** *In vitro* (18 hrs) As^+3^, As^+5^ or MMA^+3^ Exposure and Effects on Mouse Bone marrow Cell Viability^1^

Treatments	% Viability (Cellometer)	% Annexin V negative/PI negative cells	% Annexin V positive/PI negative cells	% Annexin V negative/PI positive cells
Control	63.2+1.4	78.1+2.7	16.1+2.5	5.7+0.2
5 nM As^+3^	63.3+0.6	77.8+2.2	16.3+2.1	5.8+0.8
50 nM As^+3^	58.4+3.5	76.2+1.3	17.8+0.6	5.8+0.5
500 nM As^+3^	59.8+1.7	75.6+2.0	18.4+0.4	6.0+1.7
5 nM As^+5^	59.3+0.1	77.8+1.8	16.7+0.3	5.4+1.4
50 nM As^+5^	58.8+3.7	75.9+1.4	17.1+1.5	6.7+0.4
500 nM As^+5^	62.7+2.2	74.3+0.8	19.2+1.2	6.4+0.5
5 nM MMA^+3^	59.3+3.5	76.5+1.9	17.6+2.0	5.8+0.2
50 nM MMA^+3^	59.9+6.8	78.2+0.5	16.6+0.5	5.0+0.6
500 nM MMA^+3^	39.5+10.0*	52.0+2.5*	21.6+3.4*	26.4+2.2*

1 Bone marrow cells from three mice femurs were pooled and exposed to each treatment in triplicate. Results are Means + SD with statistical significance at *p≤0.05.

**Figure 7:**
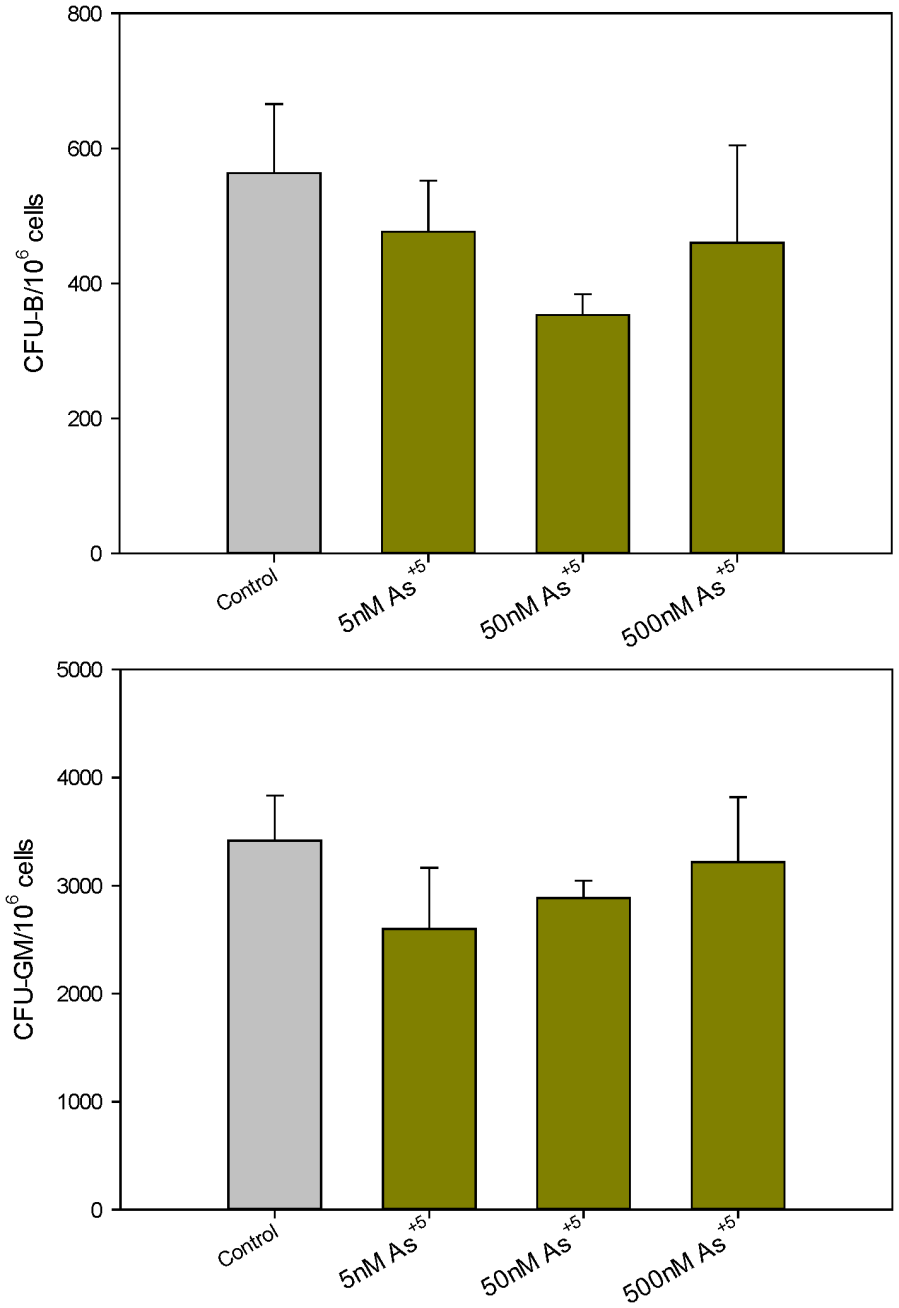
Number of colonies per million cells exposed to As^+5^ *in vitro*. Number of CFU-B colonies per million cells 10 days post plating in mouse methylcellulose media (containing As^+5^) for pre-B cells [Top]. Number of CFU-GM colonies per million cells 14 days post plating in mouse methylcellulose media (containing As^+5^) for GM cells [Bottom]. No significant differences between treatments and control (p<0.05) were found. Results are Means + SD.

Finally, since many drinking water sources are contaminated with inorganic pentavalent arsenate (As^+5^), we examined the effects of As^+5^ on mouse bone marrow progenitor cell activity *in vitro*. As^+5^ did not inhibit CFU-B or CFU-GM colony formation at concentrations as high as 500 nM (Figure 7). These results demonstrate that As^+5^ is far less toxic to the bone marrow than the trivalent arsenicals in mice.

## Discussion

Arsenic is considered to be one of the most important global environmental risk factors for human diseases [1]. There is increasing evidence that arsenic alters immune activity function in human populations. Many of the current findings rely on the use of peripheral blood and biomarkers to infer the cells and organs that are affected by arsenic [9], [17], [25], [26]. In addition, there have been *in vitro* studies with arsenite (As^+3^) at micromolar concentrations in human leukocytes that demonstrate altered immune effects possibly by affecting T cells and macrophage/dendritic cells [27], [28], [29]. Also, it has been observed that micromolar concentrations of As^+3^ induce necrosis of human CD34+ bone marrow cells [30]. These studies are interesting because arsenic trioxide is used clinically for the treatment of certain leukemias and myelodysplastic syndromes [31].

Previous studies using environmentally relevant levels of exposure have shown that low levels of As^+3^ given in drinking water to mature mice increased their susceptibility to influenza virus [7]. Our present studies followed a similar design (30 day exposure, at concentrations up to 300 ppb As^+3^). We found that As^+3^ produced a significant suppression of pre-B colony formation in the bone marrow. While the effects of As^+3^ on bone marrow could contribute to the immune suppression associated with influenza and other infections, there are numerous other immunologic abnormalities that could result from effects on the bone marrow. The hematopoietic system continually replenishes itself throughout life and is one of the most sensitive targets for cytotoxic and anti-proliferative agents, often being the dose limiting organ/tissue for radiation and chemotherapy. The immune system that develops during embryogenesis has been found to be extremely sensitive to environmental chemical effects [32]. There is evidence that the development of lymphoid tissues is altered by environmental arsenic exposure, as thymic defects have been detected in developmentally-exposed human populations [33].

To better understand the mechanisms of action associated with arsenic immunotoxicity, several labs have established animal models. Our own studies have shown that exposure of mice to arsenic trioxide via inhalation causes suppression of the T-dependent antibody response [34]. The effect of As^+3^ may involve a complex action on the activation and differentiation of both lymphoid (B and T) and myeloid (macrophages and dendritic) cells. Therefore, in the present studies, we were interested in characterizing the effects of arsenic on lymphoid and myeloid cell development in the bone marrow following *in vivo* 30 day drinking water and *in vitro* exposures. Trivalent arsenite is known to be one of the toxic species of arsenic that can form in the body following *in vivo* drinking water exposure to arsenic. The toxicity of arsenic *in vivo* would depend on the type, quantity and stability of the specific arsenic metabolites formed through biotransformation [6]. A key organic metabolite of arsenite formed through metabolism by the enzyme arsenite 3-methyltransferase (AS3MT) is MMA^+3^ [35]. To investigate the relative contribution of each arsenic species that could lead to bone marrow suppression, we directly exposed bone marrow cells to different arsenic species, including As^+3^, MMA^+3^ and As^+5^. As^+3^ significantly and selectively inhibited the lymphoid (CFU-B) progenitor formation at 50 nM, but had no effect on the myeloid progenitors (CFU-GM), even at 500 nM examined *in vitro*. The selective effects of As^+3^ on lymphoid progenitors are therefore consistent between our *in vivo* and *in vitro* studies. The lymphoid progenitor CFU-B colony formation was significantly suppressed by 5 nM MMA^+3^ *in vitro*, whereas the myeloid progenitor cells (CFU-GM), were suppressed at 500 nM MMA^+3^, indicating a hundred fold difference in sensitivity. The lack of significant changes in viability, annexin V staining and PI staining 18 hrs following *in vitro* exposure to As^+3^ (up to 500 nM), MMA^+3^ (up to 50 nM), and As^+5^ (up to 500 nM) may be an indication that cell death is not responsible for the BM suppression observed with the 300 ppb *in vivo* As^+3^ exposure. The 500 nM MMA^+3^ dose that caused the reduction in cell viability and increased annexin V and PI staining also completely inhibited CFU-B colony formation, but only suppressed CFU-GM formation by 50%. The selectivity is thus clear but raises question in regard to mechanism.

*In vitro* exposure of murine bone marrow cells to As^+5^ showed that this agent did not significantly inhibit CFU-B or CFU-GM colony formation at concentrations up to 500 nM. MMA^+3^ has been found in the blood and urine of human subjects who were exposed to high levels of inorganic arsenite via drinking water [35], [36]. It is produced in the liver and kidneys through the metabolism of arsenate or arsenite by the action of the enzymes arsenate/arsenite reductases, AS3MT, and MMA^+5^ reductase. AS3MT is a polymorphic enzyme in the human that has been shown to correlate with the toxicity of arsenic in drinking water [37], [38], [39]. AS3MT has been detected in rodent tissues, primarily in liver and kidney [40] and is also expressed in human peripheral blood [41]. Therefore, our studies demonstrate that it is important to understand the relative actions of important arsenite metabolites formed *in vivo*, such as MMA^+3^, and the exposure levels received by the bone marrow and other lymphoid organs.

In summary, this study has demonstrated for the first time the sensitivity subpopulations of the bone marrow to inorganic and organic arsenicals. The fact that lymphoid progenitors are more sensitive to suppression than myeloid progenitors suggests that there may be novel targeting mechanisms associated with cell activation and cell differentiation that are affected by As^+3^ and MMA^+3^ in murine bone marrow cells. Future studies will address various mechanisms that may be associated with arsenite inhibition of CFU-B formation and will also examine the effects of arsenite and MMA^+3^ on early T cell development.
